# A multimodal MRI study of XNKQ acupuncture for limb dysfunction after ischemic stroke: a randomized controlled study protocol

**DOI:** 10.3389/fneur.2024.1367654

**Published:** 2024-05-31

**Authors:** Chunlei Tian, Lingyong Xiao, Ruiyu Li, Yinghui Chang, Zhe Lv, Lanping Li, Shiqing Zhao, Xiaoyu Dai

**Affiliations:** ^1^Department of Acupuncture and Moxibustion, First Teaching Hospital of Tianjin University of Traditional Chinese Medicine, Tianjin, China; ^2^National Clinical Research Center for Chinese Medicine Acupuncture and Moxibustion, Tianjin, China; ^3^Imaging Department, First Teaching Hospital of Tianjin University of Traditional Chinese Medicine, Tianjin, China

**Keywords:** stroke, acupuncture, motor dysfunction, multimodal MRI, rehabilitation

## Abstract

**Introduction:**

Limb motor dysfunction is one of the challenges in rehabilitation after cerebral ischemic stroke (CIS) and greatly affects the quality of life of patients. This study aims to investigate the central mechanisms of the curative effect with multimodal magnetic resonance imaging (MRI), which will provide additional evidence to support the application of Xingnao Kaiqiao (XNKQ) acupuncture.

**Methods and analysis:**

This trial is a randomized controlled trial. Patients who meet the criteria will be recruited and randomly divided into 2 groups. One group will receive acupuncture treatment and another group will not receive acupuncture treatment. Both groups will receive conventional treatment. In addition, 20 healthy individuals will be recruited who will not receive any treatment. The total course of treatment is 14 days. The primary outcome is multimodal MRI analysis. For safety assessment, adverse events will be observed and recorded.

**Ethics and dissemination:**

The study involving human subjects was reviewed and approved by the Ethics Committee of IRB of The First Teaching Hospital of Tianjin University of TCM (TYLL2023[K]031). This study complied with the Declaration of Helsinki. Written informed consent about this study was provided by the participants. The results of this study will be published in a peer-reviewed journal.

**Clinical trial registration:**

Chinese Clinical Trial Registration Center (ChiCTR2300078315) https://www.chictr.org.cn/.

## Introduction

Data (1) from the Global Burden of Disease study show that the absolute number of stroke episodes increased by 70.0% from 1990 to 2019, while the age-standardized rates of stroke incidence decreased by 17.0%. Stroke (2) is the leading cause of disability and death in adults in China. By 2019, the number of stroke patients in China was about 28.76 million (3), of which cerebral infarction accounted for 24.18 million. If it is not treated and prevented in time, cerebral ischemic stroke (CIS) patients will be disabled or even have a recurrence of cerebral infarction in a short period, which will seriously affect their life and health and increase their economic burden.

About 12.5% of stroke survivors left with a disability in 2020 (2), equivalent to file.2 million people. After 12 months of follow-up, the rate of disability among stroke survivors was 14.8% at 3 months and 14.0% at 12 months. Therefore, how to effectively improve limb dysfunction and help patients return to daily life still needs to be further explored. In this process, exploring the brain’s mechanistic changes to improve treatment options can provide better help to patients.

Functional magnetic resonance imaging (fMRI) (4) is a non-invasive and radiation-free imaging technique, and multiple sequences can show different brain functional changes, which is widely used to study brain mechanisms (5). It can monitor the activation of brain regions, and brain functional connectivity, guide clinical rehabilitation and suggest patient prognosis.

Acupuncture is a traditional Chinese medicine treatment technique that is applied to a variety of diseases. Xingnao Kaiqiao (XNKQ) acupuncture was founded by academician Shi Xuemin of Tianjin University of Traditional Chinese Medicine. Its clinical effects have been satisfactorily verified. It is now widely used in post-stroke rehabilitation. And clinical data show its safety and efficacy. Further research is needed to support the mechanism by which XNKQ acupuncture acts on the brain in CIS patients. We used multimodal MRI technology to further explore the central mechanism by which XNKQ acupuncture exerts action in patients with post-stroke limb dysfunction.

## Methods and analyses

### Design and setting

**Figure 1:**
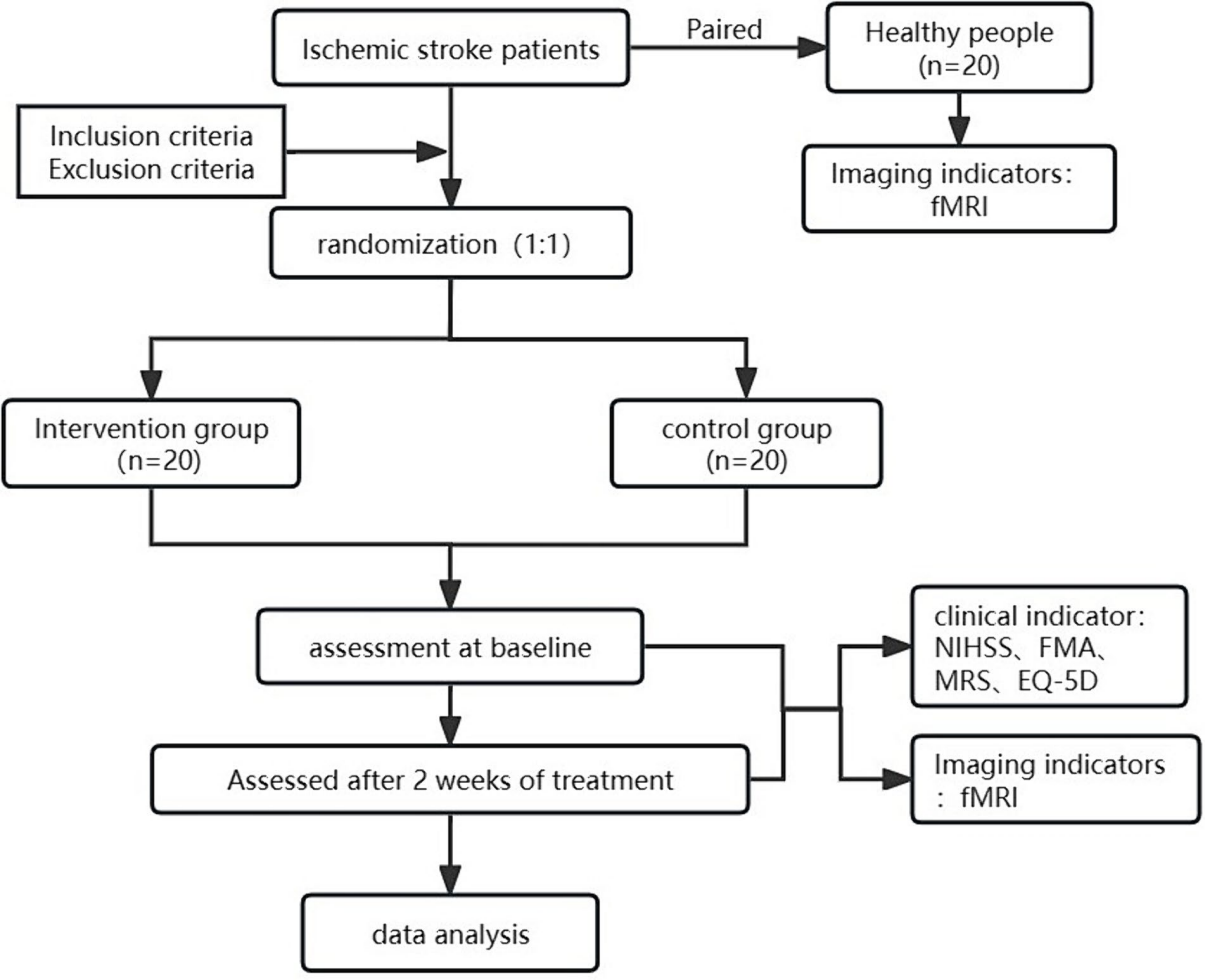
Flow chart of the trail.

This is the version of the protocol, dated 1 April 2023. The study protocol was designed in accordance with standard protocol Items: Recommendations for Interventional Trials (6) (Supplementary File 1). The study protocol was approved by the Ethics Committee of IRB of The First Teaching Hospital of Tianjin University of TCM (TYLL2023[K]031). The study will be conducted in the First Teaching Hospital of Tianjin University of Traditional Chinese Medicine. In this study, 40 patients with post-stroke limb dysfunction and 20 healthy people will be included, and patients will be randomized into 2 groups in 1:1 ratio. The intervention group will receive XNKQ acupuncture therapy and basic therapy, the control group only will receive basic therapy. Acupuncture treatment will be given 6 times a week for 2 weeks. Outcome indicators include clinical scales and imaging indicators. Healthy people will be accessed at baseline, patients will be evaluated at baseline and after 2 weeks. The flow chart of this study is shown in Figure 1. The schedules of this study is shown in Table 1.

**Table 1:** Schedule of the trial.

Time point	Study period			
	Enrollment-1 week	Allocation 0 week	Baseline 0 week	Treatment phase 2 weeks
Enrollment				
Eligibility screen	√			
Demographic	√			
Characteristics				
Informed consent	√			
Medical history	√			
Merger disease	√			
Randomization		√		
Intervention				
XNKQ group		√		
Control group		√		
Healthy People				
Assessment				
FMA			√	√
NIHSS			√	√
mRS			√	√
MBI			√	√
EQ-5D			√	√
Imaging data			√	√
Adverse event				√

### Recruitment and informed consent

Recruitment information will be available to patients in the form of posters at various locations in the hospital, and interested patients can contact the program leader by phone and e-mail. Eligible patients must meet the inclusion and exclusion criteria. If an applicant meets the study criteria, they will be invited to participate and sign written informed consent before the start of the study (Supplementary File 2). The following basic personal information will be collected: sex, age, height, weight, educational background, occupation, marital status, health insurance and related past health history, etc. These details of the participants will be for the record only and will never be revealed to any individual or organization not connected to the study.

### Diagnostic criteria

The diagnostic criteria are as follows: Refer to the diagnostic criteria for CIS in Chinese Guidelines for Diagnosis and Treatment of Acute Ischemic Stroke in 2018, all patients had acute onset and neurological deficits lasting more than 24 h. Imaging showed the presence of infarct lesions and ruled out non-vascular causes.

### Inclusion criteria

The inclusion criteria are as follows:

1. Patients group:

1. Meets the above diagnostic criteria;
2. First-ever clinical CIS occurred ≤2 weeks, the number of times ≤ 1;
3. 40–70 years old, right-handed;
4. Unilateral basal ganglia infarction and the area of infarction is 3.0 ~ 5.0 cm in diameter at the largest level, with motor dysfunction and manual muscle testing showing muscle strength grades 1 to 4;
5. No severe cognitive impairment and also mini-mental state examination (MMSE) with a score of 17 or above.
6. Provided written informed consent to participate.

2. Healthy people group

1. Age and sex were matched with the patient group, right-handed;
2. Subjects are in good health and free of organic and significant functional diseases;
3. No previous family history of the mental and nervous system;
4. No stimulant drugs have been taken in the past 2 months;
5. Provided written informed consent to participate.

### Exclusion criteria

The exclusion criteria are as follows:

1. Patients with a history of stroke, or diseases such as brain tumors or rheumatoid arthritis that result in an mRS Score of ≥2;
2. Patients with severe heart, liver, kidney, blood system, endocrine system, and mental illness, or with other diseases that prevent them from cooperating with treatment;
3. Patients with a history of metal implants in the past or with claustrophobia who are not able to finish in the MRI scanning room;
4. Persons with hearing and vision disorders that obstruct communication;
5. Pregnant and lactating women;
6. Currently participating or have participated in other clinical studies that have not yet been completed for more than 3 months.

### Removed criteria

Patients will be removed if they have:

1. Patients with poor compliance during clinical trials who voluntarily withdrew from the study;
2. Patients are not appropriate to continue the research who are with serious adverse reactions, serious complications, or deterioration of condition that occurred in the study;
3. The subjects did not complete the tasks as required (less than 50% percent of the tasks or receiving treatment of other trials) or had incomplete image observation data.

### Randomization and blinding

An independent researcher will be responsible for generating random numbers and sealed in opaque envelope. After subjects agree to participate in this study, they will randomly obtain an assigned serial number. The identification cord and basic information about each enrolled patient will be recorded on the case report form. Indicator assessors will be not aware of specific subgroups. Information exchange between patients will be not allowed. An independent researcher will perform data management and statistical analysis tasks.

### Interventions

All patients will receive the conventional basic medications which refer to the Chinese guidelines for diagnosis and treatment of acute ischemic stroke2018 (7). All of these medications will be individualized to control blood pressure, blood sugar and lipid, and prevent platelet aggregation.

The difference is that the XNKQ group will receive 30 min of acupuncture treatment. We will use stainless steel disposable acupuncture needles (size 0.25 mm × 40 mm or 0.30 mm × 75 mm) (Changchun Aikang Medical Instrument Factory) for the treatment. All acupuncture therapists have more than 5 years of clinical experience and have uniform training to ensure that the treatments will be administered consistently.

### Acupuncture group

Acupoints of XNKQ acupuncture included bilateral PC6, DU26, and affected side SP6. Patients will be in the supine position. Acupoints will be sterilized with 75% alcohol before acupuncture. The first acupuncture point to be needled will be PC6, then the needle body will be rotated to stimulate the acupoints with a speed of 40-60/min and a rotation angle of more than 180°. The second will be DU26, the needle body will be rotated 360° and fixed to the skin, and moved to stimulate the acupoint until the eye is moist or weeping. Finally, SP6 will be stimulated until the leg twitches 3 times.

Depending on each patient’s tolerance level, the therapist adjusts the acupuncture strength until the patient achieves “deqi” without significant pain. Leave the needle in place for 30 min per treatment. Acupuncture 6 times per week for 2 weeks for a total of 12 times.

### Non-acupuncture group

In the non-acupuncture group, patients will receive the same basic treatments as the acupuncture group except for acupuncture.

### Healthy group

The healthy group will not receive any interventions.

### Sample size

This study focuses on MRI data. The sample size was calculated based on similar previous studies. Through reading the research literature on the mechanism of the functional effects of acupuncture on the brain, we found that the acute phase of stroke is generally characterized by an acute condition and poor compliance. Desmond et al. (8) showed that when analyzing functional MRI data with a free threshold of 0.05, it is probably necessary to include at least 12 subjects to achieve an activation efficacy of 80% or more at the single voxel level. Anecdotal evidence (9) suggests that fMRI studies typically involve 12 to 16 subjects per group. Therefore, we included 16 subjects in each group. Considering the high dropout rate, we set the 20% dropout rate and finally included 20 subjects per group. So, a total of 40 CIS patients and 20 healthy people will be enrolled.

### Outcome measures

The study period is 2 weeks. Patients in both groups will be evaluated at baseline and week 2, the healthy individuals only at enrollment. All outcomes will be measured by evaluators who will be blinded to group allocation. All evaluators will receive the same training on these evaluations.

### Primary outcomes

Blood oxygen level-dependent functional magnetic resonance imaging (BOLD-fMRI) indirectly evaluates neuronal activity by monitoring changes in blood oxygenation (10). Amplitude of low frequency fluctuation (ALFF) and regional homogeneity (ReHo) are common evaluation indicators (11). ALFF represents the spontaneous activity of neurons, and its derivative, fraction amplitude of low frequency fluctuation (fALFF), can better remove noise signals such as heartbeat and respiration, and is more sensitive and precise. ReHo responds to intervoxel activity synchronization by calculating the consistency of voxels over time series. The two indicators have better repeatability and clear physiological significance. Functional connectivity (FC) reflects functional integration between regions.

Voxel-based morphometry (VBM) is a structural MRI quantitative analysis technique in which gray matter volume or gray matter density can be obtained by calculating the gray matter.

Diffusion tensor imaging (DTI) technique traces fiber bundle alignment and identifies structural changes in white matter fibers (12), and fractional anisotropy (FA) is the most commonly used measure reflecting the integrity of white matter fiber bundles (13).

Arterial spin labeling (ASL) is a magnetic resonance perfusion imaging technique that can be used to detect cerebral blood perfusion (14). As an important indicator, cerebral blood floot (CBF) can reflect cerebral blood flow perfusion (15).

### Secondary outcomes

National Institutes of Health Stroke Scale (NIHSS), modified Rankin Scale (mRS), Fugl–Meyer Assessment (FMA), Modified Barthel Index (MBI) and EuroQol Five Dimensions Questionnaire (EQ-5D) will be assessed before and after 2 weeks of treatment as secondary outcomes.

FMA (16) is widely used in motor function assessment which can reflect the level of limb function recovery in patients with post-stroke disability. The scale focuses on limb joint flexibility and muscle strength, including 66 points for the upper limbs and 34 points for the lower limbs, with a total score of 100 as normal. Currently, the Spanish, Russian, and German versions of the FMA are used for clinical assessment with high sensitivity and validity. Consistency of motor scores in patients with post-stroke dysfunction due to harmonization of kinematic measurement tools (17).

NIHSS (18) can quickly measure the severity of stroke and is recommended as a valid tool for stroke in the emergency department. It consists of 15 items with a total score of 42, the higher the score, the more severe it is. Numerous data demonstrate its reliability, validity, ability to predict stroke outcomes, and strong correlation with infarct foci on imaging. The NIHSS only provides a cursory assessment of the patient’s impairment and does not provide detailed information.

The mRS (19) is highly reproducible and has convergence with other disability scales. Compared to the Barthel Index, mRS is reliable in that it predicts outcomes more accurately. The World Health Organization (WHO) (20) recommends the mRS as an indicator of stroke outcome. The mRS is categorized into 7 grades covering physical function and ability to live, allowing for assessment of patient prognosis.

The MBI is a 100-point scale that assesses 10 essential abilities of patients in their daily lives, and is one of the more widely used and researched methods of assessing activities of daily living. EQ-5D has five dimensions to measure health, including mobility, self-care, daily activities, pain/discomfort, and anxiety/depression. EQ-5D-5L (21) scale which each dimension is divided into five levels, increasing the reliability, validity, and sensitivity of the measurement tool.

### Data acquisition

Data will be acquired by a Siemens Skyra 3.0 T MRI scanner (Siemens Healthineers) equipped with a 32-channel head coil. For BOLD-fMRI images, we used an echo-planar imaging (EPI) sequence (repetition time (TR) = 2,000 ms, echo time (TE) = 30 ms, flip angle = 90°, matrix = 64 × 64, field of view (FOV) = 220 mm × 220 mm, resolution = 3.4 × 3.4 × 3.0 mm^3^, gap = 0.99 mm, 240 time points, 37 interleaved axial slices). The structural image of the whole brain will be acquired for each participant using the T1 sequence (TR = 2,000 ms, TE = 1.97 ms, FOV = 256 mm × 256 mm, matrix = 192 × 192, voxel size = 1 × 1 × 1 mm^3^). White matter fiber bundle structure will be acquired from the DTI sequence (voxel size = 2 × 2 × 2 mm^3^, TE = 75 ms, TR = 8,200 ms, 70 slices, matrix = 110 × 110, FOV = 220 × 220 mm^2^, b = 0,1,000 s/mm^2^, and 64 diffusion directions). The cerebral perfusion imaging will be acquired by ASL sequence (TR = 4,000 ms, TE = 16.18 ms, matrix = 64 × 64, FOV = 220 × 220 mm^2^, voxel size = 3.4 × 3.4 × 3.0 mm^3^ TI1/TI = 700/1990 ms). Subjects’ heads will be immobilized by cushioned supports, and they will wear earplugs to protect against MRI gradient noise throughout the experiment.

For the observation steps, we will communicate with the patient in advance about the length of the scanning, so that the patient will not feel anxious if the time is too long. The subject’s ears will be plugged with cotton balls. When the patient is ready, the patient’s entire brain will be scanned. During resting-state scanning, patients will be instructed to relax, close their eyes, remain still, and avoid thinking activities as much as possible.

### Adverse events and safety monitoring

Adverse events will be assessed and managed by designated personnel within 24 h. Patients may voluntarily inform their physician of the presence of an AE at the time of the visit and during follow-up, or they may be screened for the presence of an AE by physical examination, laboratory tests, or other methods of assessment. These data will be evaluated by two investigators and documented on the CRF, including those taken by the instructing practitioner for acupuncture treatments after an AE occurs, such as discontinuance of acupuncture, resumption of acupuncture after a pause, reduction in the frequency or adjustment of the treatment regimen, continuation of acupuncture, and other interventions taken. If the adverse event is accompanied by medication or other treatment, it should be documented at the same time. Subjects may withdraw from the trial at their own discretion, or the physician may decide whether the patient should continue or terminate the study by evaluation.

### Data collection and statistical analysis

Data will be collected using the paper CRFs which will be kept in a locked cabinet at the end of the trial. Statistical analysis of data will be performed by an independent researcher. For dislocated subjects, we will record the number and reason for termination, especially adverse events. Data will be analyzed using the R software version R-4.2.2. Statistical significance is defined as a *p* value of <0.05.

For comparisons of baseline material, measurements that follow a normal distribution overall will be expressed as mean ± standard deviation ( $\bar{X}$ ± SD), and the whole data that do not follow a normal distribution will be expressed as median (interquartile range, IQR) [M (P25-P75)]. Count data will be expressed as the number of cases (n) and percentage (%) using the X^2^ test. The *t*-test or Mann–Whitney U-test will be chosen to compare the two groups’ scores on each scale based on a normal distribution and homogeneous variance. For within-group comparisons, changes in scores will be assessed by paired *t*-test or Wilcoxon rank-sum test. For subjects who discontinued or deviated from the intervention, listwise deletion or multiple interpolation will be used.

The multimodal MRI images will be analyzed by MATLAB 2023a and the statistical parameter graph software Statistical Parametric Mapping (SPM)12. Before data processing, all data will be removed the first 10 time points to retain the stabilized images; and adjust for differences arising from the time points, after which orientation alignment will be performed; and all aligned functional images will be aligned to the montreal neurological institute (MNI) space with a voxel standard size of 3 mm × 3 mm × 3 mm, and then the functional images will be smoothed with a Gaussian kernel of 6 mm × 6 mm × 6 mm. Afterward, the images will be datamined by extracting the indicators. Data will be analyzed with two independent samples *t*-tests to compare the differences between the patient group before treatment and the healthy group, as well as the differences between the acupuncture group and the no-acupuncture group after treatment. The longitudinal changes in MRI indexes before and after the respective treatments of the two groups of patients will be observed with paired *t*-tests. Age, gender, BMI, blood pressure, and history of diabetes will be used as covariates in data analysis.

Finally, the statistically significant MRI index values of the acupuncture group will be extracted and analyzed by Pearson correlation with the clinical scores of this group before and after treatment.

### Trial status

The trial will begin recruitment and treatment on 7 December 2023, and is expected to be completed by 30 December 2025.

### Quality control

In order to implement the project smoothly, we will rigorously standardize the intervention and evaluation processes. Acupuncturists with more than five years of experience will be familiar with the conduct of intervention and control group interventions to ensure consistency in the study. The assessors will be uniformly trained on the scales to ensure the reliability of the data. Ethics committee members will also review implementation throughout the trial.

## Discussion

Due to the specificity of the intervention, we were unable to achieve a double-blind design. Therefore, we design a single-blind, randomized controlled trial to evaluate the brain mechanism of the acupuncture group in the treatment of limb dysfunction in comparison to the non-acupuncture group. Patients who meet the inclusion criteria will be enrolled and randomly assigned to either the XNKQ group or the control group. Indicator assessors who are not aware of the subgroups will be objective in making indicator evaluations to avoid bias.

The fMRI is widely used in the study of brain mechanisms of acupuncture therapy for stroke. Past studies of cerebral mechanisms in patients with cerebral infarction, were focused on single acupoint such as Zusanli (ST36) and Yanglingquan (GB34). Zou et al. (22–24) studied GB34 and found that this acupoint can activate multiple sensory and motor imagery areas in patients. The FC between bilateral M1 was decreased in patients with cerebral infarction and increased after acupuncture on GB34. The researchers also found that patients tended to have increased needling effects early in the stroke period (within 1 month) and decreased responses thereafter (1–3 months). Therefore, it is necessary to intervene acupuncture treatment as soon as possible. ST36 (25) has been shown to improve post-stroke motor deficits by activating the primary motor cortex and somatosensory-motor areas. In another randomized controlled trial, ST36 (26) also increased the compensatory function of the lateral circulation in the brains of stroke patients. In addition, researchers have studied the combination of multiple acupoints and found that the combination of Baihui-Yintang-Neiguan (27) improves cerebral blood perfusion in post-stroke patients.

In this trial, we will study the brain mechanisms produced by the main point of XNKQ acupuncture in cerebral infarction patients. The XNKQ acupuncture method produces therapeutic effects by stimulating the combination of three acupoints: Neiguan (PC6), Shuigou (DU26), and Sanyinjiao (SP6). It has been widely used in the rehabilitation of stroke patients and has shown remarkable effects. In a meta-analysis of 12 randomized controlled studies of acute stroke, Yang et al. (28) found that XNKQ acupuncture significantly reduced the disability rate and improved the clinical efficacy and quality of life. In another systematic review (29), they analyzed the long-term efficacy of XNKQ and found reduced recurrence, disability, and mortality rates. Scholars also have conducted a lot of basic research (30) on acupuncture to explain the mechanism of XNKQ acupuncture. However, its brain mechanisms remain unknown. Till Nierhaus et al. (31) studied healthy individuals using the combination of PC6-DU26-SP6 and found that this combination could have an effect on functional connectivity in motor areas minutes after acupuncture. They also found that the point location only has an impact when the acupuncture points are stimulated. Based on this, we investigate the sustained effect of this combination of acupoints in stroke patients with motor dysfunction.

Zhang et al. (5) concluded that in terms of experimental design, previous research commonly used fMRI, while in terms of analytical indicators, ALFF, ReHo, and FC were more commonly used In our experiments, we will use multimodal MRI techniques to analyze brain function, also focusing on gray matter, white matter fiber tracts, and cerebral blood flow perfusion. Finally, in order to explore the predictive power of the above indexes for patient prognosis, we will evaluate the correlation of brain indicators with the clinical symptom indexes.

In summary, the results of this study will be expected to provide an argument for the mechanism of the action of XNKQ. Exploring its central mechanisms with multimodal MRI can provide an evidence-based basis for future clinical promotion.

## Ethics statement

The study involving human subjects was reviewed and approved by the Ethics Committee of IRB of The First Teaching Hospital of Tianjin University of TCM (TYLL2023[K]031). This study complied with the Declaration of Helsinki. Written informed consent about this study was provided by the participants. The investigators will report any changes of protocol or informed consent to the Ethics Committee and Registry.

## Author contributions

CT: Methodology, Writing – original draft. LX: Writing – original draft. RL: Methodology, Project administration, Writing – review & editing. YC: Methodology, Writing – review & editing. ZL: Project administration, Writing – review & editing. LL: Project administration, Writing – review & editing. SZ: Project administration, Writing – review & editing. XD: Resources, Funding acquisition, Methodology, Writing – review & editing.
